# Policy-practice gaps and barriers to the effective management of insecticide resistance in Tanzania

**DOI:** 10.1186/s12936-026-05983-0

**Published:** 2026-06-08

**Authors:** Hamenyimana E. Gervas, Irene R. Moshi, GloriaSalome G. Shirima, Ismail H. Nambunga, Rukiyah M. Njalambaha, Sheikha S. Mohamed, Rosemary P. Nshama, Charles D. Mwalimu, Maranya M. Mayengo, Yeromin P. Mlacha, Prashanth Selvaraj, Fredros O. Okumu, Halfan S. Ngowo

**Affiliations:** 1 Nelson Mandela African Institution of Science and Technology (NM-AIST), P.O. Box 447, Arusha, Tanzania; 2 Environmental Health and Ecological Science Department, Ifakara Health Institute, P.O. Box 53, Ifakara, Tanzania; 3 Department of Mathematics and Statistics, University of Dodoma, P.O. Box 338, Dodoma, Tanzania; 4 Institute for Disease Modeling, Global Health Division, Gates Foundation, Seattle, Washington United States of America; 5 School of Biodiversity, One Health and Veterinary Medicine, University of Glasgow, Glasgow, G12 8QQ UK; 6 National Malaria Control Programme (NMCP), Dodoma, Tanzania

## Abstract

**Background:**

Malaria control gains are stagnating or reversing, in part due to widespread insecticide and drug resistance, as well as increasing financial and implementation challenges. This study assessed the national strategies for insecticide resistance monitoring and management in Tanzania to identify key barriers and misalignments between policy and actual practice.

**Methods:**

We initially reviewed technical documents from Tanzania’s National Malaria Control Programme (NMCP) produced between 2014 and 2024, along with World Health Organization (WHO) guidelines and reports on insecticide resistance monitoring and management. This was followed by in-depth interviews with key informants in Tanzania, including policymakers, representatives from funding agencies, technical experts, scientists, and vector control implementers at district and regional levels. The qualitative data was analyzed thematically using NVivo software.

**Findings:**

The document review revealed strong policy alignments of the NMCP strategies for monitoring and management of insecticide resistance with international practice as recommended by WHO. However, implementation of the policy intentions remained limited, with resistance monitoring being conducted in only 22 of Tanzania’s 184 district councils. Interviews with 24 stakeholders highlighted significant gaps between the stated policies and guidelines and the actual practice. These gaps were driven by inadequate financing, donor dependence, insufficient coordination and dissemination of guidelines, limited technical capacity, and weak engagement of communities and district-level operators. Participants emphasized that without strengthened surveillance systems, sustainable local financing, and greater community and intersectoral collaboration, achieving the current strategic goal of malaria elimination by 2030 remains unlikely.

**Conclusion:**

Despite strong policy alignment and strategic planning, Tanzania’s capacity to implement insecticide-resistance management initiatives remains constrained by several policy-practice gaps, particularly in funding, staffing and coordination. Addressing these persistent challenges will be essential for more effective malaria vector control and will require sustained financing, stronger coordination, improved vector monitoring, and greater community and intersectoral engagement.

## Background

Although malaria is preventable and treatable, it remains a major health challenge in Africa, disproportionately affecting the poor and marginalized populations [1]. Despite substantial progress driven by interventions such as insecticide-treated nets (ITNs), indoor residual spraying (IRS), effective diagnosis and treatment, preventive therapies, and more recently, vaccines [2–8], this progress has slowed, and in some areas stalled or even reversed [1, 9]. This stagnation is driven by multiple factors, including rising commodity costs amid dwindling resources, increasing insecticide and drug resistance, inadequate adherence of the affected population to the interventions, and suboptimal intervention coverage, underscoring a widening gap between current progress and the global malaria control targets [3].

In Tanzania, malaria vector control has historically been predominated by insecticide-based approaches complemented with environmental sanitation [10–13]. For decades, these efforts were organized through local health systems, until the National Malaria Control Programme (NMCP), established in 1990 [10, 14], began reinforcing nationally coordinated and regional initiatives [10, 13]. From the early 1990 s, ITN delivery strategies were piloted in Bagamoyo and Kilombero, leading to nationwide ITN scale-up starting in the late 1990 s [10, 14]. IRS was relaunched in 2007, initially covering two districts and later expanding to 18 councils in three regions of the north-west and Lake zone, using pyrethroids at first, followed by bendiocarb, and from 2014 onward, organophosphates [10, 14–16]. However, in 2020, IRS was done in only six districts with the highest malaria burden regions in the same zone [17].

Over 60 million ITNs were distributed between 2009 and 2020, with continued large-scale distribution through antenatal care and immunization programs, schools, and mass campaigns [10, 14]. Because of these efforts, and the expanded case management [18, 19], malaria prevalence among children fell from 14% in 2016 to 7.5% in 2017 [10, 18, 19]. However, this progress later reversed, with prevalence increasing to 8.1% in 2022, and rates exceeding 15% in the Lake, Western, and Southern Zones [18, 19]. Separately, pyrethroid resistance expanded markedly over time, with the proportion of sentinel sites reporting resistance increasing from 0% in 2004 to more than 80% by 2020 [17, 20]. Although this overlap may suggest a possible association between increasing pyrethroid resistance and the resurgence of malaria transmission, other contributing factors, including disruptions in malaria surveillance and health service delivery during the COVID-19 pandemic [3], may also have influenced the observed increase in prevalence. Nevertheless, widespread pyrethroid resistance among *Anopheles* mosquitoes remains a major concern for the effectiveness and long-term sustainability of pyrethroid-based vector control interventions in Tanzania, potentially undermining progress toward malaria elimination [21–23].

Tanzanian public health authorities have made several efforts to address these insecticide resistance challenges, aligning with global and regional practices [24–29]. The NMCP strategic plans and guidelines have consistently included actions to manage insecticide resistance, informed by both local evidence and international recommendations [10, 17, 30]. These frameworks serve as implementation roadmaps, emphasizing strategies such as insecticide rotation for IRS, combined use of ITNs and IRS with different active ingredients, the application of insecticide mixtures and mosaics to enhance vector control [10, 30–32], and more recently the deployment of new generation ITNs that include either insecticide synergists or multiple active ingredients [2, 3, 32], and the scale-up of larval source management (LSM) across the country [10, 33].

Despite well-structured plans, implementing interventions to achieve targeted goals has historically been challenging [10, 26, 30], with success requiring context-specific strategies and solutions unique to Tanzania. For instance, delays in ITN distribution reported in strategic plans [10, 30] are not simply due to negligence or a lack of commitment; rather, they underscore the inherent difficulty in translating strategic goals into effective implementation. Such delays, particularly in replacing standard pyrethroid-only ITNs with next-generation ITNs in areas with widespread pyrethroid resistance, may prolong reliance on less effective nets, potentially exacerbating resistance and undermining the effectiveness of vector control interventions. Critical questions remain about the discrepancies between insecticide resistance monitoring and management (IRM) policies and their on-the-ground implementation, the barriers hindering effective IRM strategies, and the resulting constraints on malaria elimination efforts. Consequently, Tanzania's prospects for achieving nationwide malaria elimination in the near future remain uncertain.

This current study aimed to critically assess the national strategies for insecticide resistance monitoring and management in Tanzania, with the objective of identifying key barriers and misalignments between policy and actual practice. To achieve this, we used a combination of an in-depth review of key technical documents underpinning IRM practices in Tanzania and internationally, alongside an analysis of the opinions and perspectives of key stakeholders in vector control activities in the country.

## Methods

### Study design

*Document review:* This study was conducted between February and November 2024 and commenced with a comprehensive document review aimed at assessing national and international policies, strategies, and guidelines on insecticide resistance monitoring and management (IRM) over the past decade (2014–2024). The review focused on documents produced by Tanzania’s NMCP, the Ministry of Health (MoH), the World Health Organization (WHO) and malaria control partners. The identification was done through systematic searches of the government and WHO official websites, complemented by searches in PubMed, Google Scholar databases and other scientific repositories. Search terms included combinations of keywords such as “malaria vectors”, “insecticide resistance,” “vector control”, “insecticide resistance management,” “insecticide resistance monitoring” and “entomological surveillance”. Eligible documents included national reports, strategic plans, guidelines, frameworks, and evaluation documents relevant to malaria vector control and resistance management. For WHO documents, those published before 2010 were excluded.

Key national policy documents were retrieved from the MoH and NMCP websites, including two NMCP Strategic Plans (2014–2020 and 2021–2025) [10, 30], the Integrated Vector Management Guidelines and Standard Operating Procedures (IVM-SOP) [32], the National Guidelines for Integrated Malaria Vector Control (NGIMVC) [34], the Malaria Surveillance, Monitoring and Evaluation Plan (MSMEP) [35], and the National Strategy for Vector Control 2019–2024 (NSVC) [36]. Searches of WHO databases and scientific repositories identified several complementary global publications, including the Global Plan for Insecticide Resistance Management in Malaria Vectors (GPIRM) [31], the Manual for Monitoring Insecticide Resistance in Mosquito Vectors and Selecting Appropriate Interventions [37], the Global Report on Insecticide Resistance in Malaria Vectors [38], and the Framework for a National Plan for Monitoring and Management of Insecticide Resistance in Malaria Vectors [39]. The full list of documents reviewed is included in Table 1.

**Table 1:** List of documents reviewed to understand current policies, guidelines and practices of insecticide resistance monitoring and management in Tanzania’s malaria control program

Document title	Organization	References
National Malaria Strategic Plan 2014–2020	NMCP, Tanzania	[30]
National Malaria Strategic Plan 2021–2025	NMCP, Tanzania	[10]
Integrated Vector Management Guidelines & Standard Operating Procedures	MoH, Tanzania	[32]
National Guidelines for Integrated Malaria Vector Control (NGIMVC)	NMCP, Tanzania	[34]
Malaria Surveillance, Monitoring & Evaluation Plan (MSMEP)	NMCP, Tanzania	[35]
National Strategy for Vector Control 2019–2024 (NSVC)	MoH, Tanzania	[36]
Tanzania Malaria Indicator Surveys	MoH, Tanzania	[18, 19]
National Social and Behavior Change and Advocacy Guide for Malaria Interventions 2021–2025	NMCP, Tanzania	[40]
Tanzania (Mainland) Malaria Profile FY 2023	U.S. PMI, Tanzania	[41]
Tanzania (Mainland) Malaria Operational Plan FY 2023	U.S. PMI, Tanzania	[42]
Global Plan for Insecticide Resistance Management in Malaria Vectors (GPIRM)	WHO	[31]
Manual for Monitoring Insecticide Resistance in Mosquito Vectors and Selecting Appropriate Interventions	WHO	[37]
Test procedures for insecticide resistance monitoring in malaria vector mosquitoes (Second edition)	WHO	[43]
Global Report on Insecticide Resistance in Malaria Vectors	WHO	[38]
Guidelines for Malaria Vector Control	WHO	[44]
Framework for a National Plan for Monitoring and Management of Insecticide Resistance in Malaria Vectors	WHO	[39]
World Malaria Reports 2019–2024	WHO	[1, 9, 45–48]

*U.S. PMI* denotes the United States President’s Malaria Initiative, *WHO* the World Health Organization, *MoH* the Ministry of Health, *NMCP* National Malaria Control Programme. All documents were publicly available online at the time of access, were downloaded, and are available from the corresponding author upon request if no longer accessible online

*Key informant interviews:* To complement the document review, key informant interviews were conducted with policymakers, representatives of funding agencies, field implementers, and technical experts. The policy-level interviewees included representatives of key government agencies, including the NMCP, the President’s Office-Regional Administration and Local Government (PO-RALG), the National Institute for Medical Research (NIMR), the Tanzania Plant Health and Pesticides Authority (TPHPA), operating under the Ministry of Agriculture (MoA). Others included representatives of non-governmental organizations, namely US President’s Malaria Initiative (PMI), Population Services International (PSI) and the WHO. District and regional level field implementers interviewed included those based in Mwanza, Kigoma, and Tabora regions, where the country was most recently implementing both ITNs and IRS strategies.

A total of 24 key informant interviews were conducted, either in person or virtually (via Zoom) for participants who were unable to join in-person meetings. Participants were purposively selected to ensure representation of all key stakeholder groups directly involved in insecticide resistance management and malaria control efforts. Of the participants, 18 were males and 6 were females; and all were between 30 and 55 years old, and had a tertiary education. The discussions were held primarily in Swahili (Tanzania’s main language) with occasional use of English. The semi-structured interview guide comprising 27 open-ended questions was used to explore participants’ understanding and perceptions of insecticide resistance, insecticide resistance monitoring and management (IRM) strategies, implementation challenges, and gaps between policy and practice. The flexible interview format allowed for probing and follow-up questions tailored to participants’ expertise and responses. Ethical approval for the entire study process was obtained from the Institutional Review Board (IRB) of Ifakara Health Institute and the National Institute for Medical Research (NIMR).

Each interview lasted between one to two hours and was audio-recorded with participants’ permission. Recordings were supplemented with detailed field notes to capture contextual observations. Written informed consent was obtained from all participants before data collection.

### Data analysis

All audio recordings were transcribed word-for-word and thoroughly reviewed to ensure accuracy. To maintain the authenticity and contextual integrity of participants’ responses, transcripts were retained in original language used during the interview (Swahili). The qualitative data were analyzed using thematic content analysis, employing both deductive and inductive approaches to identify pre-defined major themes and emerging themes. NVivo software (version 15) [49] was used to organize and manage codes, themes, and patterns. The major themes identified included: knowledge and perceptions of insecticide resistance, views on resistance monitoring and surveillance systems, insecticide resistance management strategies, implementation challenges, and discrepancies between policy and actual practice.

## Results

### Insights from document review

#### Alignment of national strategies with global IRM frameworks

The review of national and international documents revealed that Tanzania has progressively aligned its strategic direction with global frameworks for insecticide resistance monitoring and management (IRM) over the past decade. The NMCP strategic plans (2014–2020 and 2021–2025) [10, 30] define the core pillars of Tanzania’s malaria agenda, including integrated vector control; malaria diagnosis, treatment, and preventive therapies; and surveillance, monitoring, and evaluation. These plans are guided by the national vision of a malaria-free society and a mission to ensure equitable access to high-quality, effective, and affordable malaria services through partnerships and community participation.

In support of this vision, a suite of policies, frameworks, and technical guidelines have been developed, namely, the Integrated Vector Management Guidelines and Standard Operating Procedures (IVM-SOP) [32], the National Guidelines for Integrated Malaria Vector Control (NGIMVC) [34], the Malaria Surveillance, Monitoring and Evaluation Plan (MSMEP) [35], and the National Strategy for Vector Control 2019–2024 (NSVC) [36]. Collectively, these documents are consistent with WHO recommendations and key global guidance documents, including the Global Plan for Insecticide Resistance Management in Malaria Vectors (GPIRM) [31], the Manual for Monitoring Insecticide Resistance in Mosquito Vectors [37], the Global Report on Insecticide Resistance in Malaria Vectors [38], and the Framework for a National Plan for Monitoring and Management of Insecticide Resistance in Malaria Vectors [39].

#### Integrating of the principles of the global plan for insecticide resistance management (GPIRM)

Overall, the reviewed documents indicate that Tanzania has adopted the major pillars of the GPIRM [31], adapting them to national priorities and operational contexts. In particular, the planning and implementation of IRM strategies, together with systematic surveillance and monitoring of insecticide resistance, are recognized as critical components of the national response. The reviewed frameworks underscore the urgent need for coordinated, proactive, and sustained action to address the increasing threat of resistance, especially in high-burden areas such as the northern, southern, and south-eastern regions of the country as well as the districts bordering Lake Victoria in the north and northwest [18, 19, 41].

Another area of alignment with global recommendations was that Tanzania’s national IRM framework also emphasizes both immediate and long-term interventions such as insecticide rotations, combinations, mosaic spraying, and the use of insecticide mixtures [32]. More recently, the incorporation of next-generation tools, including piperonyl butoxide (PBO)-ITNs and innovative insecticide formulations for IRS, reflects the country’s commitment to maintaining vector susceptibility and prolonging the efficacy of available insecticides. We observed that Tanzania constantly updates its guidelines to match the WHO pronouncements, and that both the international and national documents constitute the foundation for the planning, execution, and evaluation of integrated vector control interventions.

Lastly, the document review found strong alignment with WHO’s standardized guidelines for insecticide resistance monitoring [31], which advocate the use of validated bioassays, prioritization of key vectors and insecticides for testing, and adaptation of control interventions based on resistance data [32, 35, 36].

#### Gaps between policy and implementation

Despite this strong policy alignment, the review identified persistent gaps between strategic intent and implementation outcomes. The reviews highlight that resource limitations, logistical challenges, and operational inefficiencies continue to hinder full realization of planned activities [10, 30]. It was noted that only 22 of the 62 sentinel districts chosen for malaria vector surveillance have been used to monitor insecticide resistance [10], which makes the national resistance profile less representative.

These shortfalls were attributed to delays in ITN distribution, high operational costs of the operations, inadequate financing, and gaps in human and infrastructural capacity, all of which collectively result in suboptimal intervention coverage and limited protection for at-risk populations, potentially undermining malaria control efforts.

### Insights from in-depth key informant interviews

This section presents insights from key informant interviews with policymakers, representatives of funding agencies, technical experts, and district- and regional-level field implementers from Mwanza, Kigoma, and Tabora regions.

#### Knowledge and perception on insecticide resistance

Most respondents had a good understanding of insecticide resistance and its impact on malaria control. Some linked it to antimicrobial resistance, and others said that the use of pesticides in farming was a major cause of resistance because the agricultural pesticides are of similar classes to public health insecticides. However, regarding resistance levels and spread in Tanzania, most respondents were unable to provide specific information, citing a lack of accessible statistical data. The quotes below provide further insights:

“*Pesticides used in agriculture are also applied in public health, exposing mosquitoes to the same insecticides. This repeated exposure can cause the development of insecticide resistance in vector populations…*” (Male participant, district-level field implementer).

*“… yes, insecticide resistance is a growing problem. It was once limited to a few areas but has now spread. Although I don’t have enough data, but the problem is serious”* (Male participant, regional-level field implementer).

#### Insecticide resistance monitoring and management

According to the expert respondents, insecticide resistance monitoring in Tanzania began in 1999, initially focusing on testing the susceptibility of primary malaria vectors to pyrethroids using WHO insecticide susceptibility assays. Over time, the programme has expanded from a few sites to 22 out of 184 sentinel councils nationwide, with a strategic shift to prioritize districts with high malaria prevalence. However, most respondents noted that this coverage is not nationally representative, making it difficult to generalize the findings to the entire country:

*“…if we had at least one monitoring site in each region, it would improve the representativeness. We can’t reach every area, but covering more councils would give a better national picture. Right now, we talk about representation, but it’s difficult to claim that 22 councils represent the situation in all 184…”* (Male participant, technical expert)

*“...currently, we have about 184 or 185 councils, if I’m not mistaken, but resistance monitoring is conducted in only 22. I believe this coverage is too small to adequately represent the insecticide resistance problem across the entire country”* (Female participant, representative of funding agency).

#### Discrepancy in policy and practice

Nearly all respondents stated that there were discrepancies between policy and actual practice. They attributed these gaps to factors such as funding and resource constraints, coordination challenges, and shortages of trained personnel. For example, although Tanzania’s current policy recommends deploying multiple interventions using insecticides with different modes of action, such as rotating organophosphates and neonicotinoids for IRS, the stakeholders interviewed here indicated that these stipulated strategies have not been effectively implemented. It was also stated that while insecticide rotations had been the most widely practiced strategy historically, specifically in the districts receiving IRS, this was no longer the case as the practice of IRS had been significantly reduced in Tanzania:

*…yes, I can say that there is a clear discrepancy between policy and actual practice in the field, largely due to limited resources. The government depends heavily on external funding; as a result, certain interventions, such as IRS, cannot be implemented as prescribed in the policy, particularly after donor support for IRS was withdrawn* (Male respondent, policymaker).

*“Rotation was the most used method, but now it is combination. This was possible due to IRS, but with IRS gone, rotation is no longer used, though it was very effective”* (Male participant, district-level field implementer).

#### Concerns related to limited financing as a barrier to effective implementation of insecticide resistance monitoring and management strategies

Nearly all respondents identified inadequate financing as a primary barrier to effective implementation. Many noted that although ITNs are intended to be replaced every 3 years, logistical challenges and funding shortfalls frequently delay distributions, extending replacement cycle:

*“…our policies are well-designed and clearly articulated; however, the main challenge lies in the lack of funding to implement what is outlined in the policy”* (Female respondent, policymaker).

*“…the main challenge is funding. If NMCP had its own allocated budget from the government, clearly defined with set amounts for specific needs like net procurement, I don’t think there would be such difficulty in implementation”* (Male respondent, technical expert).

“*You order the insecticide (biolarvicides) to arrive during the dry season when breeding sites are easily identifiable, but the funds are disbursed during the peak of the rainy season*” (Male respondent, district-level field implementer).

The respondents also emphasized that, the country is highly depending on donor funds to ensure availability of malaria prevention interventions. They noted that such dependency affects the availability on interventions but also impacts on the selection of appropriate interventions, which they argued is more likely to reflect donor’s and not country preference:

*“…but most delays are due to reliance on external funding sources, which come with their own bureaucratic procedures that must be followed before funds are released. That’s where the discrepancies arise*” (Male respondent, regional-level field implementer).

“*…much of the fight against malaria is donor-funded, if I'm not mistaken, since around 1999. I wonder what our situation will be if they will fully withdraw their support...”* (Male participant, technical expert)

*“One among the challenges for not being able to implement what is indicated within the policy is presence of a lot of priorities at regional and district levels, which inhibit allocation of enough funds for malaria prevention activities”* (Female respondent, district-level field implementer).

Majority of respondents highlighted the need to mobilize domestic resources to address current funding gaps. They also recommended active engagement of local stakeholders and allocation of revenues from national natural resources, such as mining sectors to strengthen investments in vector control:

“*We have abundant natural resources, including mines like Geita Gold Mining and Lakes. Why are we not allocating revenue from these resources to support malaria elimination and resistance management efforts?”* (Male participant, regional-level field implementer).

*“I believe internal funds exist in abundance; it's just a matter of priority. For example, a single wealthy individual and banks like CRDB and NMB can spend a lot of money sponsoring football leagues. I'm not saying it's not important; it is, but the same funders could also be encouraged to support public health initiatives. So why aren’t they being engaged in malaria prevention and elimination efforts*?” (Male participant, technical expert).

#### Concerns related to poor coordination and shortage of trained personnel

Respondents identified several additional challenges, including bureaucratic delays in fund disbursement to local councils and poor coordination of roles and activities. In Tanzania, although policies are formulated at the national level, implementation occurs at the council level, where guidelines are often distributed but insufficiently disseminated, leading to limited ownership among implementers. Some participants noted overlapping or unclear responsibilities between the Ministry of Health (MoH) and the President’s Office-Regional Administration and Local Government (PO-RALG): the MoH develops policies and strategic plans, while PO-RALG oversees implementation. This structural arrangement has resulted in sub-optimal engagement of district-level operators, fund misallocation, staffing challenges, and a top-down approach that limits input from local implementers. This has also contributed to delays in execution of insecticide resistance management activities.

“*The main challenge causing discrepancies is the communication gap between national policymakers and council-level implementers. Plans are made at the top, but implementation happens at the grassroots...”* (Male respondent, regional-level field implementer).

*“…I would say the guidelines are distributed but not effectively disseminated, which cause some local implementers to lose their sense of ownership…”* (Female respondent, district-level field implementer).

*“…for example, I assessed the larvicide needs in my council, including coverage and costs, totaling nearly 22 million TZS. However, I was later informed during a regional meeting that only 4.5 million TZS was available. I was left wondering how to proceed...”* (Male respondent, district-level field implementer).

Another concern was the lack of formal recognition for personnel trained at the Vector Control Training Centre (VCTC) in Tanga, whose salaries remain at volunteer level despite completing 2–3 years of specialized training. Accordingly, respondents stressed that, alongside regular training and proper coordination, which are essential for effective insecticide resistance management, policymakers should actively disseminate guidelines and provide context-specific training to local implementers:

*“...regular trainings are essential and I suggest that those staff who attended the training at our college in Tanga (Vector Control Training Centre), should be considered for appropriate salary scale categorization to enhance their motivation, …every district should have a dedicated vector control specialist, such as an entomologist, to strengthen vector control efforts at the district level, unlike the current situation”* (Male participant, district-level field implementer).

#### Inadequate community malaria education, social media engagement, and community participation

Respondents identified insufficient community-based malaria education as a key factor widening the policy-practice gap. Participants stressed that malaria elimination requires strong community engagement, effective use of social media platforms, and culturally tailored behavior change communication strategies. Educated communities are more apt to sustain preventive practices, such as using insecticide-treated nets and managing mosquito breeding sites. Additionally, involving religious and political leaders as advocates was considered vital for community mobilization, perception shifting, and widespread adoption of malaria prevention and resistance management strategies:

*“…. to ensure successful elimination, we should also invest in behavior change design and campaigns aimed at altering community perceptions on malaria and interventions, tailored to the specific characteristics of each region..”* (Male participant, regional-level field implementer).

*“Once a person is educated, even the interventions you plan to introduce become much easier to implement and use. Therefore, in the fight against malaria, we must invest significantly in educating the community. I believe that if the community is fully educated, the malaria problem can be reduced to more than 50%”* (Male participant, technical expert).

#### Perceptions on the current malaria control targets

When asked about the adequacy of current strategies and tools to address insecticide resistance and achieve malaria elimination by 2030, most respondents expressed skepticism. The discontinuation of IRS and limited support for biolarviciding were viewed as major barriers to sustaining progress.

Participants agreed that achieving elimination targets will require reduced dependence on external funding and increased domestic resource mobilization. They emphasized the need to elevate malaria control and IRM to national priorities, comparable to the country’s response to other major diseases such as HIV/AIDS. Strengthening intersectoral collaboration, particularly among the Ministry of Health (MoH), Ministry of Agriculture (MoA), and the Ministry of Infrastructure, was also identified as essential to advancing national malaria elimination efforts.

*“… eliminating malaria by 2030 is like a dream; let’s be honest. It will only be possible with blanket larviciding; otherwise, it remains just a dream. Even regions with less than 1% malaria prevalence are simply benefiting from colder climates that are unfavorable for mosquito breeding”* (Male participant, regional-level field implementer).

“*Due to the withdrawal of the IRS and the lack of donor support for biolarviciding, achieving the goal of reducing transmission by 90% and mitigating resistance will be highly challenging*” (Female participant, technical expert).

## Discussion

This study examined the alignment between national policies and on-the-ground implementation of insecticide resistance management strategies in Tanzania’s malaria control ecosystem. It critically evaluated national insecticide resistance monitoring and management (IRM) strategies and identified key barriers and policy-practice gaps. Although the NMCP has established comprehensive frameworks consistent with WHO recommendations, substantial implementation challenges remain. These challenges stem from limited resources, constrained surveillance coverage, reliance on external funding, and weak coordination across administrative levels. While these challenges are not unique to Tanzania, similar obstacles are widely documented across malaria-endemic countries in sub-Saharan Africa, where the transition from policy formulation to effective operationalization remains a major bottleneck in resistance management and vector control [24, 27, 50–53]. The findings from this study highlight the urgent need for stronger domestic financing, improved multisectoral collaboration, and sustained community engagement to ensure that national policy commitments translate into measurable progress toward malaria elimination.

Persistent funding and resource constraints, coupled with heavy donor dependence, pose major challenges to effective IRM and malaria elimination. Over the past decade, more than two-thirds of malaria control investments in endemic countries have relied on external funding. In Tanzania, approximately 90% of malaria financing is provided by the Global Fund (GF) and the President’s Malaria Initiative (PMI), contributing an estimated USD 265 million during 2021–2023 [10]. However, a funding gap exceeding USD 200 million remains for the 2021–2025 strategic plan [10]. While these partnerships have enhanced program efficiency and targeted resource allocation [4, 54–56], dependence on external financing creates vulnerabilities, particularly when procurement and tendering processes delay the delivery of key interventions [25, 57]. This was evident in delays in the distribution of ITNs [10], including the replacement of standard ITNs with next-generation ITNs. Such delays are especially concerning in areas with widespread pyrethroid resistance, where next-generation ITNs play a critical role in maintaining vector control effectiveness [10, 30]. Given that the effective lifespan of an ITN is typically less than 3 years [7], delayed replacement reduces household access to functional and insecticide-effective nets. As nets age, their physical integrity and insecticidal efficacy decline [7], increasing human exposure to infectious mosquito bites and potentially contributing to sustained malaria transmission. Consistent with prior studies highlighting the pivotal role of internal financing in malaria control [24, 58–60], this study emphasize the need to strengthen domestic resource mobilization to reduce vulnerability to fluctuations in external support, particularly amid recent donor funding cuts from agencies such as USAID [10, 58, 61]. Although domestic resources may not fully replace international contributions in the short term [62], increased national investment could enhance IRM implementation by expanding surveillance and monitoring activities and supporting complementary vector control interventions, such as IRS and larviciding, which have often been constrained by limited funding [31, 38].

Beyond funding, enhanced community engagement and education, improved coordination, adequate human resources, and effective decentralization of health services are critical for successful malaria control interventions [60, 63–66]. Our study underscores that engaging influential community figures, such as religious leaders, local chiefs, and politicians, can strengthen IRM implementation and advance malaria elimination goals. Consistent with previous evidence [65, 67–70], community acceptability and participation are pivotal to the success of interventions, as they cultivate local ownership and sustainability. Ensuring that communities are meaningfully engaged throughout planning, implementation, and dissemination phases allows their priorities and concerns to be addressed, which enhances acceptance and uptake [71]. This is particularly important given ongoing efforts by the NMCP to expand knowledge and awareness of malaria and its interventions within Tanzanian communities [40]. Furthermore, robust intersectoral collaboration, strengthened institutional coordination, and a well-trained health workforce equipped with continuous professional development and policy guidance are equally vital for sustaining effective IRM implementation [27, 72–74].

This study aimed to provide additional insights to support ongoing IRM strategies and was not intended to alter NMCP or partner efforts in malaria control. It acknowledges the success of national policies emphasizing routine resistance monitoring, evidence-based vector control, and insecticide rotation aligned with global IRM frameworks [32, 34, 35]. The national monitoring system, despite covering few sites, has produced high-quality resistance data, demonstrating the NMCP’s strong commitment to surveillance [10, 32, 35]. Similarly, insecticide management strategies, including rotating non-pyrethroid insecticides for IRS and transitioning to next-generation ITNs, have successfully restored mosquito susceptibility in several regions [10, 30]. However, implementation has been inconsistent, particularly following IRS withdrawal and recent funding reductions [75–78]. This study highlights the urgent need to address implementation barriers to ensure effective insecticide resistance management and malaria control. Consistent with existing evidence on the role of non-pyrethroid interventions in managing resistance [79–84], the findings also underscore the importance of reintroducing non-pyrethroid IRS where appropriate and fully implementing complementary measures such as larviciding. The summary of recommendations (Table 2) is proposed to strengthen IRM implementation and accelerate malaria elimination in Tanzania.

**Table 2:** Summary of recommendations to strengthen insecticide resistance management and malaria elimination efforts in Tanzania

Challenge	Recommendation(s)
Funding and resource constraints, and donor dependence	Increase domestic funding and resource mobilization through public–private partnerships, improved taxation, health levies, innovative financing mechanisms (including diaspora bonds), and efficient use of natural resource revenues. In particular, diaspora bonds can provide an additional source of financing by enabling Tanzanians living abroad to invest in national health and development priorities, including malaria control and insecticide resistance management Enhance governance, transparency, and accountability, while reducing corruption
Poor coordination and shortage of trained personnel	Establish a well-defined coordination unity for insecticide resistance monitoring and management (IRM) with a clear mandate and dedicated budget Disseminate guidelines and provide context-specific training to local implementers Establish adequate and equitable remuneration structures for vector control personnel to enhance motivation, retention, and workforce capacity
Inadequate community malaria education and engagement	Strengthen community engagement and culturally tailored behavior change communication by involving community, religious, traditional, and political leaders, leveraging social and mass media platforms, and integrating malaria education into school curricula

Despite the novelty of this study, its findings should be interpreted within the context of its scope and design. The study focused specifically on challenges and policy-practice gaps in the implementation of vector control strategies related to insecticide resistance monitoring and management in Tanzania; therefore, the findings may not be generalizable to other health issues, disease control programs, or sectors. Furthermore, the analysis reflects policies and implementation practices at a particular point in time and may not fully capture recent policy developments or ongoing initiatives. Although the number of in-depth interviews was relatively small and participants were drawn from a limited number of regions, efforts were made to include a diverse range of key stakeholders, including policymakers, funding partners, technical experts, and implementers at regional and district levels. These participants were considered well-positioned to provide informed perspectives on the issues under investigation. Nevertheless, future studies could strengthen the evidence base by including a larger and more geographically diverse sample, particularly among district- and regional-level implementers. In addition, the document review was limited by the availability of publicly accessible policy and programmatic documents. However, the inclusion of key documents from the NMCP and the WHO enhanced the contextual relevance and validity of the findings.

## Conclusions

This study provides compelling evidence that, although Tanzania’s malaria control frameworks align with global best practices, their implementation remains constrained by persistent operational and systemic challenges. These include funding limitations, overdependence on donor support, limited technical and operational capacity, weak coordination, low community engagement, insufficient intersectoral collaboration, and sociocultural barriers. Such challenges may hinder progress toward malaria elimination targets. Strengthening insecticide resistance management will require sustained financing, improved coordination, expanded surveillance systems, and greater community and multisectoral engagement. Prioritizing these actions is critical not only for effective resistance management but also for realizing the national goal of a malaria-free Tanzania.

## Data Availability

Data are available from the corresponding author(s) upon reasonable request.
